# Treatment of Mode Coupling in Step-Index Multimode Microstructured Polymer Optical Fibers by the Langevin Equation

**DOI:** 10.3390/polym14061243

**Published:** 2022-03-19

**Authors:** Svetislav Savović, Linqing Li, Isidora Savović, Alexandar Djordjevich, Rui Min

**Affiliations:** 1 Center for Cognition and Neuroergonomics, State Key Laboratory of Cognitive Neuroscience and Learning, Beijing Normal University at Zhuhai, Zhuhai 519087, China; savovic@kg.ac.rs (S.S.); linqingli07@mail.bnu.edu.cn (L.L.); 2 Faculty of Science, University of Kragujevac, R. Domanovića 12, 34000 Kragujevac, Serbia; 3 Laboratory of Neurodegenerative Disease, School of Biomedical Sciences, Li Ka Shing Faculty of Medicine, The University of Hong Kong, 21 Sassoon Road, Pokfulam, Hong Kong, China; u3008169@connect.hku.hk; 4 Department of Mechanical Engineering, City University of Hong Kong, 83 Tat Chee Avenue, Kowloon, Hong Kong, China; mealex@cityu.edu.hk

**Keywords:** microstructured polymer optical fiber, Langevin equation, mode coupling

## Abstract

By solving the Langevin equation, mode coupling in a multimode step-index microstructured polymer optical fibers (SI mPOF) with a solid core was investigated. The numerical integration of the Langevin equation was based on the computer-simulated Langevin force. The numerical solution of the Langevin equation corresponded to the previously reported theoretical data. We demonstrated that by solving the Langevin equation (stochastic differential equation), one can successfully treat a mode coupling in multimode SI mPOF as a stochastic process, since it is caused by its intrinsic random perturbations. Thus, the Langevin equation allowed for a stochastic mathematical description of mode coupling in SI mPOF. Regarding the efficiency and execution speed, the Langevin equation was more favorable than the power flow equation. Such knowledge is useful for the use of multimode SI mPOFs for potential sensing and communication applications.

## 1. Introduction

**Figure 1:**
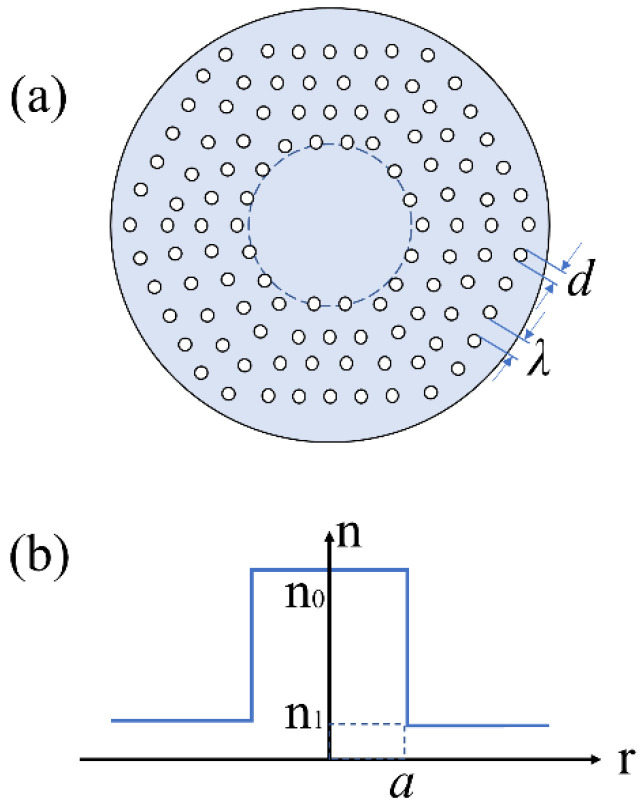
(**a**) Cross-section of multimode SI mPOF, (**b**) refractive-index profile of the referent multimode SI mPOF.

Compared with silica optical fibers, polymer optical fibers (POFs) have a larger diameter (up to 1 mm) and can be easily paired with VCSELs and LEDs, although the transmission losses of POFs are higher [1]. POFs are good candidates for short-range transmission systems such as automotive and home network connections [2,3]. Moreover, due to their advantages such as a large negative thermo-optic coefficient, high bending flexibility, and large elastic strain limits, POFs are promising for sensing applications [4,5,6,7,8,9]. A microstructured optical fiber or photonic crystal fiber (PCF) was first demonstrated in 1996 with silica material [10], which can realize a wide variety of properties by different microstructures with a solid core and a hollow core [11,12]. This technology is transferable from silica material to other materials so the first non-silica microstructured optical fiber reported was made of the polymer [13]. The change to polymers had advantages due to the wide range of polymer materials and low processing temperature [14,15,16]. As an alternative to standard optical fibers, a profile of the refractive index (RI) of the microstructured optical fiber can be adjusted by selecting an appropriate material and hole pattern in the cladding [17]. The RI distribution and numerical aperture of the SI mPOF can be adjusted by varying fiber design parameters. For example, holes that are uniform in size can form a regular triangular lattice over the SI mPOF cladding (Figure 1). Thus, the central part without holes has the highest RI while the effective value of the RI of the cladding n_1_ can be easily reduced with larger or more densely spaced holes in the cladding. The transmission of light along the microstructured optical fibers is influenced by differential mode coupling, modal attenuation, and modal dispersion [18]. Mode coupling is the process of energy transfer between neighboring modes during their propagation along the optical fiber. Mode coupling is mostly induced by intrinsic random perturbations of the fiber, such as refractive index variations, microbends, and stresses [19,20,21].

The angular input optical power distribution that results from a specific launch gets modified gradually with distance from the input fiber end by the effect of mode coupling. The expected beam properties, including the far-field radiation pattern, are altered as a consequence [19,20]. Thus, for example, if we arrange a centrally symmetric launch (along a cone) at a fixed angle *θ = θ*_0_ to the fiber axis, a ring can be imaged behind the output end of a short fiber—the ring diameter is related to that initial launch angle $\theta_{0}$. As the fiber is “lengthened” (replaced by longer and longer fibers), the edges of this ring become blurred and the ring morphs gradually into a disk. This is due to effects of mode coupling accumulating with distance from the input end and causing the angular power distribution, initially narrowly centered around *θ = θ*_0_, to gradually widen and shift towards *θ* = 0°. At the coupling length *L_c_*, the distribution, even of the highest order guiding mode, has shifted its midpoint to zero degree, where the equilibrium mode distribution (EMD) is achieved. By lengthening the fiber to beyond the value known as $z_{s}$, the angular light distribution becomes fixed and centered (the disk is brightest in its center). This is a steady-state distribution (SSD) that is independent of the launch conditions except for the overall brightness: normalized to its peak value, the SSD is one and the same whatever the launch angle(s). By employing the power flow equation [19], these patterns have been predicted as a function of the launch conditions and fiber length in SI mPOF. In this paper, we report for the first time on the application of the Langevin equation in the treatment of mode coupling in SI mPOF. This way, by solving a stochastic differential equation (the Langevin equation), we show that one can successfully treat a mode coupling in multimode SI mPOF caused by its intrinsic random perturbations.

## 2. The Langevin Equation

We have previously reported that the Langevin equation can be employed in the investigation of mode coupling in standard step-index plastic optical fibers [22]. In this work, we investigate the state of mode coupling along the SI mPOFs by employing the Langevin equation. The Langevin equation can be written as [23]:

(1) $$\frac{d\theta }{dz}=W+g\Gamma \left(z\right)$$

where *z* is the distance from the end of the fiber’s input, *θ* is the propagation angle measured with respect to the optical fiber core axis, *W* is the drift coefficient and gΓ(z) is a random Langevin force with the strength g, and where:

〈Γ(z)〉=0

(2) $$\langle \Gamma \left(z\right)\Gamma \left(z^{\prime }\right)\rangle =2\delta \left(z-z^{\prime }\right)$$

The Langevin Equation (1) can be expressed in the following form [23]:

(3) $$\frac{d\theta }{dz}=W+\sqrt{D}\Gamma \left(z\right)$$

where *D* is the mode coupling coefficient. It should be noted that the second term in Equation (2) represents the intrinsic perturbation effects of the fiber’s internal noise which has a stochastic nature. The process described by the Langevin Equation (3) with the δ-correlated Langevin force (2) is known as a Markov process, i.e., its probability distribution at length *z_n_* depends only on the value *θ_n−_*_1_ at the preceding position *z_n−_*_1_. To solve the Langevin Equation (3), the fiber length *z = z_f_* is divided into *N* length steps *k*:

(4) $$z_{n}=kn;\;k=\frac{z_{f}}{N};\;n=1,\;2,\ldots ,\;N$$

Then, the angle *θ_n+_*_1_ at fiber length *z_n+_*_1_ is determined by the following discretized Langevin equation:

(5) $$\theta_{n+1}=\theta_{n}+Wk+\sqrt{Dk}\omega_{n}$$

where *n* = 0, 1,…, *N* − 1 and *ω*_0_, ω_1_,…, *ω_N−_*_1_ are independent random numbers with Gaussian distribution, zero mean <*ω_n_*> = 0, and variance <*ω_n_ ω_n′_*> = 2δ*_nn′_*. This way, one obtains *θ_N_* = *θ*(*z_f_*). By calculating a large number of representations of *ω_n_*, and averaging in appropriate intervals ∆*θ*, one obtains <*θ*(*z_f_*)>. 

One should mention here that it is well known that perturbations in an optical fiber are random in nature, and they include density and concentration fluctuations, microscopic random bends caused by stress, diameter variations, and fiber core defects such as microvoids, cracks, or dust particles. The power flow equation [19,20] is deterministic in nature, and it does not describe the energy redistribution in an optical fiber as a stochastic process caused by fiber perturbations. Since the Langevin equation is stochastic in nature, the stochastic process of energy redistribution in an optical fiber caused by its perturbations is explicitly described and modeled by the Langevin equation.

## 3. Numerical Results and Discussion

By solving the Langevin equation, we study the influence of mode coupling on transmission in a solid-core multimode mPOF. The following two equations for effective parameter *V* are used to calculate the effective RI of cladding $n_{fsm}$ for SI mPOFs [24,25]:

(6) $$V=\frac{2\pi }{\lambda }a_{eff}\sqrt{n_{0}^{2}-n_{fsm}^{2}}$$

(7) $$V\left(\frac{\lambda }{\Lambda },\frac{d}{\Lambda }\right)=A_{1}+\frac{A_{2}}{1+A_{3}\exp\left(A_{4}\lambda /\Lambda \right)}$$

where *n*_0_ is the RI of the core, Λ is the pitch, *d* is the hole diameter of the cladding,$\;a_{eff}=\Lambda /\sqrt{3}$ [25], λ is the operating wavelength, and fitting parameters $A_{i}$ (i=1 to 4) are given as:

(8) $$A_{i}=a_{i0}+a_{i1}{\left(\frac{d}{\Lambda }\right)}^{b_{i1}}+a_{i2}{\left(\frac{d}{\Lambda }\right)}^{b_{i2}}+a_{i3}{\left(\frac{d}{\Lambda }\right)}^{b_{i3}}$$

The coefficients $a_{i0}$ to $a_{i3}$ and $b_{i1}$ to $b_{i3}$ (i=1 to 4) are given in Table 1.

**Figure 2:**
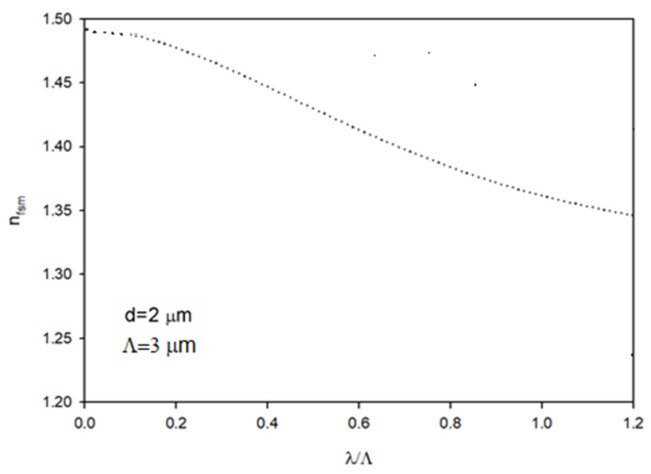
Effective RI of the cladding as a function of λ/Λ.

Figure 2 depicts the effective RI of the cladding $n_{1}\equiv n_{fsm}$ as a function of λ/Λ, for pitch Λ=3 μm and the hole diameter of the cladding d=2 μm. The effective RI is $n_{1}$ = 1.4458, the core RI is $n_{0}$ = 1.492 and the relative RI difference is ∆ = $(n_{0}-n_{1})/n_{0}$ = 0.691611 (operating wavelength is λ = 645 nm). The coupling coefficient for this fiber was assumed to be *D* = 1.649$\times 10^{-4}$ rad^2^/m [19]. In the calculations, we used a drift coefficient *W* = (0.0051 ± 0.0005) rad/m, which was determined by averaging the rate of switching from the ring to the disk output field pattern for low- and high-order modes (incidental angles) [22]:

(9) $$W=\left(\frac{1}{M}\right)\overset{M}{\underset{r=1}{\sum }}W_{r}$$

where *W_r_* is a drift coefficient of the *r*th mode. In Equation (9), drift coefficients *W_r_* (*r* = 1, 2) for modes with launch angles *θ*_0_ = 5°and 10°were averaged. We performed a Monte Carlo sampling of $5\times 10^{5}$ representations of the *ω_n_* in Equation (5) in intervals ∆*θ* = 0.2°, where *k* = 0.0001 m was used.

**Figure 3:**
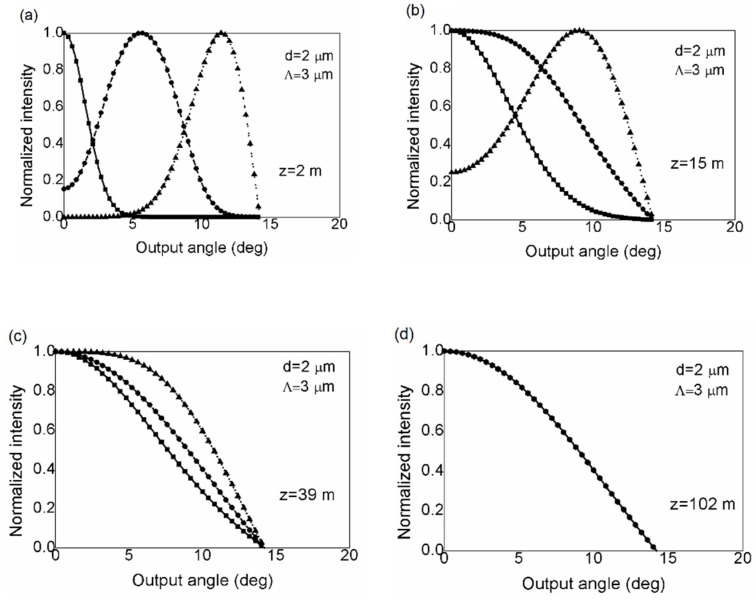
Normalized output angular power distribution calculated by solving the Langevin equation for launch angles $\theta_{0}$ = 0°(∎), 5°(•), and 10°(▲) and normalized output angular power distribution calculated by solving the power flow equation [19] for launch angles $\theta_{0}$ = 0°(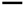), 5°(- - -), and 10° (• • •), for fiber length (**a**) *z* = 2 m; (**b**) *z* = 15 m; (**c**) $z\equiv L_{c}=39\;m$; (**d**) $z\equiv z_{s}$ = 102 m (Λ=3 μm and *d* = 2 µm).

In Figure 3, we show the evolution of the normalized output angular power distribution with the fiber length for SI mPOF, which is our numerical solution to the Langevin equation. The results shown in Figure 3 for three different input angles *θ*_0_ = 0°, 5°, and 10° are compared with our previously reported results obtained by solving the power flow equation [19]. There is a high degree of agreement between these results, with mean square errors below 1%. The radiation patterns in the short fiber (*z* = 2 m) in Figure 3b indicate that distributions of low-order modes have shifted towards *θ* = 0°. Higher-order mode coupling can be observed after longer fiber lengths. It is not until a fiber’s coupling length *L_c_* of 39 m that all the mode distributions have shifted their midpoints to zero degree (from the initial value of *θ*_0_ at the input fiber end), producing the EMD in Figure 3c. The coupling continues beyond the *L_c_* mark until all distribution widths equalize and SSD is reached at length *z_s_* in Figure 3d: *z_s_* = 102 m.

Regarding the efficiency and execution speed, the Langevin equation is more favorable than the power flow equation. In contrast to the power flow equation where a very fine mesh in the finite difference method is needed in order to achieve a high accuracy of the numerical solution, there is no such problem with the Langevin equation. The efficiency of the algorithm for integration of the Langevin equation and algorithm for obtaining the numerical solution of the power flow equation using an explicit finite difference method was measured in terms of time efficiency (execution speed) and complexity (structure of the solution/algorithm). For the largest analyzed fiber length of 102 m, the execution time on an Intel^®^ Core™ i3 CPU 540 at 3.07 GHz computer for the Langevin equation and the power flow equation was 1.8 min and 2.7 min, respectively. The numerical solution of the power flow equation is more complex than the solution of the Langevin equation.

Finally, the importance of knowing the coupling length *L_c_* lies in the fact that at fiber lengths shorter than *L_c_*, the pulse spreading is linear with length, while after establishing the EMD at length *L_c_*, it has a *z*^1/2^ dependence. Therefore, the shorter length *L_c_* is more desirable since it results in a slower bandwidth decrease [26].

## 4. Conclusions

By employing the Langevin equation (stochastic differential equation), we investigated the influence of mode coupling on transmission characteristics of the SI mPOFs. The numerical solution of the Langevin equation corresponded to the previously reported theoretical data. It is important to note that the Langevin equation, which is a stochastic differential equation (in contrast to the power flow equation, which is deterministic in nature), recognizes and explicitly accounts for the stochastic nature of the fiber’s intrinsic perturbation effects which cause mode coupling. Regarding the efficiency and execution speed, the Langevin equation is more favorable than the power flow equation. In contrast to the power flow equation where a very fine mesh in the finite difference method is needed in order to achieve a high accuracy of the numerical solution, there is no such problem with the Langevin equation. Our numerical results obtained by solving the Langevin equation compared with our previously reported results obtained by solving the power flow equation for the analyzed SI mPOF showed a high degree of agreement, with mean square errors below 1%. Mode coupling influences the fiber’s bandwidth in such a way that the sooner the EMD is achieved the faster a bandwidth improvement in SI mPOFs occurs. Namely, at fiber lengths shorter than the coupling length *L_c_* the pulse spreading is linear with length, while after establishing the EMD at length *L_c_*, it has a *z*^1/2^ dependence. Therefore, the shorter length *L_c_* is more desirable since it results in a slower bandwidth decrease in SI mPOFs. This is significant because mode coupling has an impact on the vast majority of fiber-based applications.

## Figures and Tables

**Table 1:** Fitting coefficients in Equation (8).

	i=1	i=2	i=3	i=4
$a_{i0}$	0.54808	0.71041	0.16904	−1.52736
$a_{i1}$	5.00401	9.73491	1.85765	1.06745
$a_{i2}$	−10.43248	47.41496	18.96849	1.93229
$a_{i3}$	8.22992	−437.50962	−42.4318	3.89
$b_{i1}$	5	1.8	1.7	−0.84
$b_{i2}$	7	7.32	10	1.02
$b_{i3}$	9	22.8	14	13.4

## Data Availability

The data presented in this study are available on request from the corresponding author.
